# Dynamic Involvement of Real World Objects in the IoT: A Consensus-Based Cooperation Approach

**DOI:** 10.3390/s17030484

**Published:** 2017-03-01

**Authors:** Virginia Pilloni, Luigi Atzori, Matteo Mallus

**Affiliations:** 1 Department of Electrical and Electronic Engineering (DIEE), University of Cagliari, Cagliari 09123, Italy; matteo.mallus@diee.unica.it; 2 GreenShare Srl, Cagliari 09128, Italy; matteo.mallus@green-share.it

**Keywords:** resource allocation, Internet of Things, virtual objects

## Abstract

A significant role in the Internet of Things (IoT) will be taken by mobile and low-cost unstable devices, which autonomously self-organize and introduce highly dynamic and heterogeneous scenarios for the deployment of distributed applications. This entails the devices to cooperate to dynamically find the suitable combination of their involvement so as to improve the system reliability while following the changes in their status. Focusing on the above scenario, we propose a distributed algorithm for resources allocation that is run by devices that can perform the same task required by the applications, allowing for a flexible and dynamic binding of the requested services with the physical IoT devices. It is based on a consensus approach, which maximizes the lifetime of groups of nodes involved and ensures the fulfillment of the requested Quality of Information (QoI) requirements. Experiments have been conducted with real devices, showing an improvement of device lifetime of more than 20%, with respect to a uniform distribution of tasks.

## 1. Introduction

In the Internet of Things (IoT) vision, even the most common and simple object is expected to acquire information from the surrounding ambient and to cooperate with other objects to achieve a common application goal, fulfilling the expected quality requirements. This is the case for instance of different cars that are moving in a given urban area that is affected by different congestion points and that can share the knowledge about the status of the roads so that they can better find the route that minimizes the driver objects, typically expressed in terms of expected time to reach the destination. They can also share information about the parking lots occupancy so as to reduce the time needed to park the car. Another situation is the one of devices that are located in the same geographical area, either indoor or outdoor, and share the knowledge about the temperature so that the IoT applications can benefit from a more accurate view of this physical magnitude. Indeed, many are the use-cases where the devices can collaborate in the implementation of a specific operation of either sensing or actuation for the benefit of the applications that exploit the relevant services. Clearly, the devices need to implement a certain level of logic to coordinate in the execution of collaborative actions so as not to deplete the precious battery energy doing operations performed by other devices uselessly. Accordingly, the resulting scenario of interest for this paper can be defined as the one where a group of devices have in common the capability to perform the same tasks (e.g., sensing the temperature in a given geographical area, measuring the traffic status in a road), which entails for a procedure to decide about their involvement when an application requires the execution of this task. Important aspects to be considered herein are: the consumption of the energy and its impact on the network and devices lifetime; the Quality of Information (QoI), which measures the characterisation, in terms of some salient attributes represented in the form of metadata, of the goodness of the data collected, processed and flowing through a network on the basis of a specific user’s need at a specific time, place, physical location, and social setting [1,2]. Collaborative approaches in this scenarios are also fostered by the widespread adoption of cloud computing technologies to augment capabilities of simple and cheap devices to take part in the deployment of complex applications [3], especially through the introduction of the Virtual Object (VO) [4] concept, which is the digital counterpart of a physical entity. Accordingly, the collaboration among devices is typically implemented by the VOs, following either a centralized or decentralized approach. According to the former, the procedure runs in the cloud, which needs to be constantly updated about the status of the objects. According to the latter, the devices directly interact each other and agree on the best solution without the involvement of the central platform.

In this paper, we focus on the decentralized approach and propose a consensus-based sensing allocation algorithm, which has a twofold objective: (i) considering QoI constraints in the process of allocating tasks to the IoT objects, so that the fulfillment of application requirements is ensured; and (ii) optimizing the use of resources of the underlying IoT system by maximizing the lifetime of the group of devices involved. Experiments have been conducted with real devices, showing that we can reach an improvement of lifetime of more than 20% and 60%, with respect to the cases of a uniform distribution of tasks and task assignment to the lowest energy-consuming device, respectively. The convergence time has also been proved to be quite fast in the range of 200 ms per task.

The paper is organized as follows. Section 2 presents reference works on resource allocation in the IoT. Section 3 provides a description of the reference architecture and of the problem addressed. Section 4 describes the proposed consensus-based solution for resource allocation in the IoT and a computational complexity analysis. Section 6 provides simulations and experimental results. Finally, conclusions are presented in Section 7.

## 2. Past Works

The IoT consists of intelligent objects connected to the Internet, which cooperate to support the execution of complex applications and services [5]. These objects are equipped with sensors and actuators, which provide context-awareness and enable them to gather, process and exchange data, in order to react to external stimuli. The need to represent, store, discover, search, exchange and manage the huge amount of information generated by the objects, motivated the development of semantic technologies [6].

In the past few years, many well-known IoT middleware architectures based on virtualisation of real objects have been proposed [4]. The management and resource allocation of these objects is usually committed to the power of cloud computing, which ensures high reliability, scalability and autonomy to provide ubiquitous access, dynamic resource discovery and composability of application tasks [3,7]. According to [8], the physical components of an object can be abstracted and made available as virtual resources. Meaningful virtualisation models of physical devices can be found in the Wireless Sensor Networks (WSNs) field, as described in [9,10,11]. Virtualisation allows the higher layers of the IoT architecture to: (i) interface with devices; (ii) provide devices with the required commands, adapted to their native communication protocol; and (iii) monitor their activities and connection capabilities. The result of virtualisation, i.e., the VO, is defined by [12] as the virtual counterpart of one or more real objects, and, as such, it inherits all their functionalities, characteristics and acquired information. In [13], the authors propose a framework for sensor Cloud in a Smart City context, to enable the necessary resources, storage and computing capabilities for large amounts of heterogeneous and personalized data coming from distributed sources in a transparent and secure manner. However, reaching the cloud to manage network nodes is not always a good solution, especially for real-time applications, nor is it a convenient solution in terms of energy consumption. This issue is partially solved in [14], where fog computing is used to virtualise real world object characteristics and resources, and to allocate application tasks to them.

Resource allocation has been extensively studied in WSNs, particularly with reference to network lifetime. In [15], a distributed task allocation that focuses on the reduction of the overall energy consumption and task execution time into a heterogeneous WSN is proposed, with attention to nodes’ residual energy. A similar approach is studied in [16], where a distributed algorithm based on particle swarm optimization is proposed. Since the main criticality of wireless networks is their lifetime, all of these algorithms mainly focus on maximizing this resource. Nevertheless, IoT nodes have more heterogeneous characteristics and capabilities, including residual memory, processing capacity and throughput.

As far as IoT networks are concerned, distributed resource allocation is an open issue. Most of the existing studies on resource allocation for IoT are focused on IoT service provisioning, such as in [17,18]. In these studies, the aim is to allocate the resources that enable service execution. However, they do not focus on finding the best configuration that corresponds to an optimal resource allocation. None of the works found in the literature tries to find the optimal resource allocation associated to the lowest impact of the application assigned to the network. Additionally, QoI is not taken into account [19].

## 3. Reference Scenario and Problem Statement

**Figure 1:**
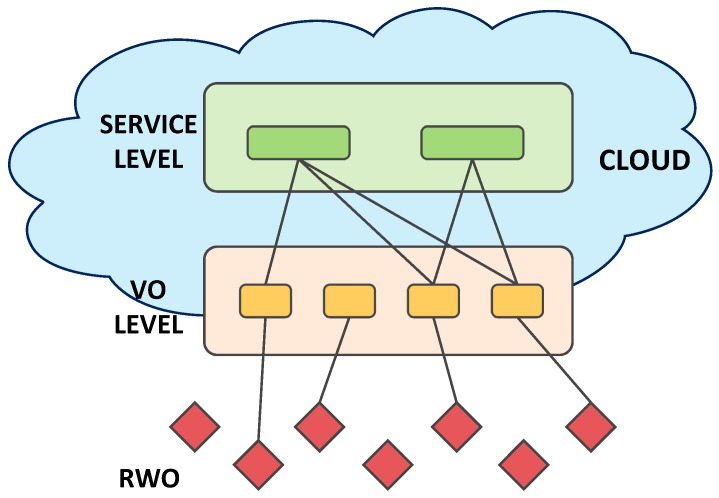
The reference IoT cloud architecture.

We refer to the Cloud-based IoT model that relies on virtualization technologies [20] and includes three levels, as shown in Figure 1. A Real World Object (RWO) is a device that has the ability to observe the real world phenomena and to perform measurements or operate on other objects. The Virtual Object [4] is its digital representation and guides its involvement in the implementation of the deployed IoT applications, by providing a description of the RWO with also semantic enrichment. It also supports discovery and a mash up of services, improving the objects’ energy management efficiency, as well as addressing heterogeneity and scalability issues. Each VO is instantiated with a template that should match the type of RWO to which it is associated (e.g., smartphone model, embedded device type, temperature sensor), along with the functionalities that it is able to provide. A new VO template is instantiated anytime a new RWO is discovered by the system, and its characteristics are changed dynamically when any possible RWO’s change is experienced (e.g., new geographic location, a change in the amount of available resources, and new functionalities provided). Depending on the RWO capabilities and use-cases, the VO processes are run in the cloud, gateway or RWO physical devices. Scenarios where the VO functionalities are distributed among these locations are also possible. The Service Level receives user application requests, translates them in computer language and sends them to the VO level, which maps them dynamically to the appropriate VOs, which take charge of their accomplishment by involving the relevant RWOs.

These deployment processes need to take into consideration the applications’ QoI. QoI is the characterisation, in terms of some salient attributes represented in the form of metadata, of the goodness of the data collected, processed and flowing through a network [1]. QoI concerns the information that meets a specific user’s need at a specific time, place, physical location, and social setting. Some examples of QoI requirements are data sampling rate, precision, and provenance [2].

**Figure 2:**
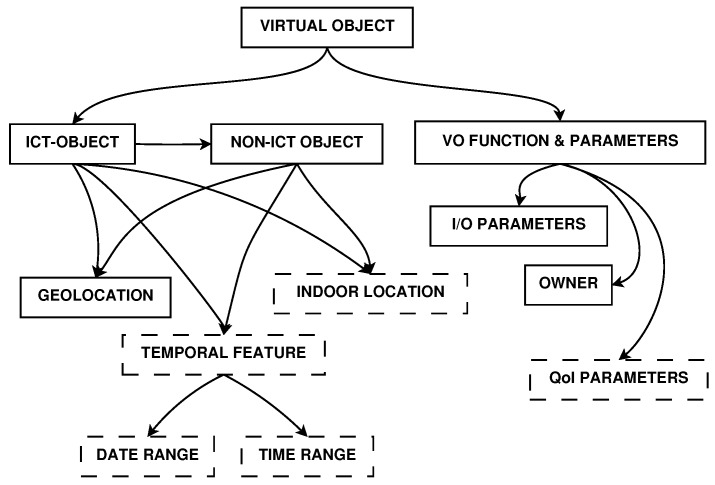
Virtual Object Information Model used. Solid border boxes correspond to elements included in the iCore VO Information Model. Dashed border boxes are new elements introduced by the proposed architecture.

An important component of this architecture is the Information Model, which is implemented by the VO template and encodes all the information that is used for their appropriate involvement in the IoT application deployment and delivery. A great effort in the definition of the Information Model has been done by the iCore FP7 project [21]. However, we needed to extend this model to be effective for our target, and we modified it, taking into account the mobility of objects, their temporal features and their characteristics of QoI. This enhancement is meant to improve the VO search, discovery and selection processes that enable the task’s assignment to the most appropriate VOs, with a QoI-oriented perspective. Figure 2 shows the new elements in dashed border boxes:

- **Indoor Location**: It is particularly useful in cases of closed environments. This could be an element that enhances the scalability of the system. It permits the model to be used not only in large-scale distributed environments (metropolitan areas or neighbourhoods), but also in small size environments and internal locations (such as buildings or structures in which a geo-localization of the nodes is not enough).
- **Temporal Features**: The use of the temporal features, both in terms of date and time range, allows for knowing the activity phases of a device associated with its VO. Knowing the date and time in which a mobile device is located in a given place and helps the association process among ICT (Information and Communications Technology) and non-ICT objects. It also ensures the ability to know in advance when a particular resource is available, when it is possible to refer to it, and how long it has not been updated.
- **QoI Parameters**: The Information Model, on which the selection processes are based, includes a field dedicated solely to the QoI parameters. The values in this field are named uniquely based on their characteristics. In addition, it introduces their descriptive aspects that allow their identification. The parameters stored in this field will therefore be examined in the selection phase and allow an optimised choice of the resources to use.

When a new application has to be deployed, the service level sends a request to the VO level to search, among the available VO instances, those that are able to perform the relevant tasks based on the appropriate templates and other parameters (e.g., position, ownership). To this, the Information Model becomes vital to implement an effective search function. As a result, for each requested task *k*, a group of VOs capable of performing it are identified. At this point, there is the need to decide how they should contribute to the execution of this task while considering the required QoI level. In the following, we consider that the target QoI level is a required execution frequency $F_{k}^{ref}$. However, the proposed solution can be generalized to other QoI requirements.

**Figure 3:**
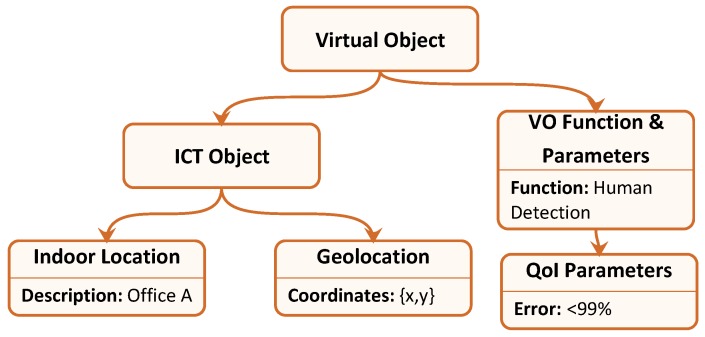
VO Information Model required to respond to the query coming from the Service Level, in the reference example.

In order to explain the process more clearly, we introduce an explanatory example: we suppose that the service level receives a request for the evaluation, every minute, of human presence inside office *A*, in the building located at coordinates {*x*,*y*}, with an error no higher than 99%, minimising processor usage. Therefore, the service level sends this request to the VO level, which analyses the request and determines the VO Information Model that can respond to it. Hence, the VO level starts searching for VO instances characterised by an Information Model with parameters equal to those described by Figure 3. Suppose that the VO level finds three VOs that match the queried one, which correspond to the following RWOs: a PIR (Passive Infra Red) sensor, which captures human movements; a camera, which uses pattern recognition to detect human faces; and a thermal sensor, which detects body heat. The fact that the same service can be provided by such heterogeneous devices using so different functionalities, is completely transparent to the VO level, which can manage all of them simply by managing their VOs and related attributes. At this stage, the VO level sends a request for human presence detection to one of the VOs, including also the required frequency $F^{ref}=1/60$ Hz, and the resource to minimise, i.e., processor usage. The VOs can then start reaching consensus using the approach described in Section 4, regardless of their localisation with respect to their related RWO.

### 3.1. Location of the Virtual Objects

**Figure 4:**
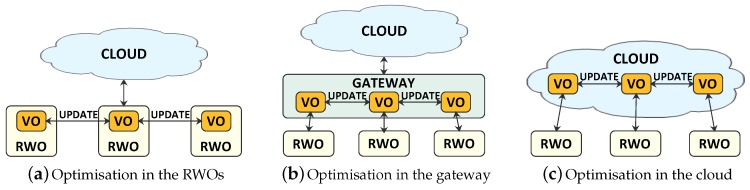
Location of the proposed algorithm into three typical IoT scenarios with reference to objects’ resource allocation.

The decentralized approach introduced in our proposal has the advantage of being able to better follow the changes of RWOs’ status. This is possible when the VO task allocation functionalities are implemented in the RWOs. We base on the consideration that nodes that are assigned to the same task are usually located close to each other, and thus they can be able to form a connected group relying on short-range communication technologies, as shown in Figure 4a. In this scenario, RWOs reside in the same area and are characterised by sufficient computational power and energy. The optimisation process can be distributed on RWOs, in such a way that the management of resources is as close as possible to the point where they are used.

In case RWOs do not have sufficient computing power, the VO task allocation functionalities are implemented in the gateways, and fog/edge computing technologies are used [22] (Figure 4b). Since we are considering devices that are connected to local networks, gateways can take charge of VOs’ functionalities and run the optimisation process, involving all the RWOs interested in the connection. Once VOs reach consensus on the gateway, they send the resulting execution frequency to their RWOs.

In both cases, the optimisation mechanism does not pass through the Internet network, i.e., it does not introduce overhead outside of the local network, and it is faster, characterised by lower latency, less expensive from an energy point of view, and incurs in no connectivity issues.

If the devices selected to perform the task are not in the same area, but they are located in different places and at a great distance, their communication can take place only through the Internet. In this case (Figure 4c), the optimisation process is carried out in the Cloud. Although the Cloud is characterised by higher resources, communication between VOs and RWOs has to pass through the Internet. Since VOs have to be frequently updated about the status of RWOs’ resources, having VOs located remotely from RWOs would increase the amount of resources needed to synchronise them. Furthermore, higher latency is experienced. Therefore, the first two solutions are preferable.

## 4. The Resource Allocation Model

### 4.1. Resource Model

In this paper, we focus on the main resources that represent an issue for IoT systems: object lifetime, storage capacity, and processor and data throughput.

#### 4.1.1. Lifetime

As defined in [23], the lifetime of a node is defined as the time until it depletes its battery. Applying this to our case, the lifetime of the node associated to VO *i* at time *t* is

(1) $$\tau_{i}^{lftm}\left(t\right)=\frac{E_{i}^{res}\left(t\right)}{\sum_{k}E_{ik}^{c}\cdot f_{ik}\left(t\right)}$$

where $E_{i}^{res}\left(t\right)$ is its residual energy, $E_{ik}^{c}$ is the energy consumed by the RWO associated to VO *i* to perform task *k*, and $f_{ik}\left(t\right)$ is the frequency at which VO *i* performs task *k*. This means that the lifetime of a node depends on the frequency at which the tasks assigned to it are performed.

#### 4.1.2. Storage Capacity

The storage capacity of a node decreases according to the frequency at which data are stored in it and to the amount of data stored. Analogously to the definition of node lifetime, we define the storage capacity depletion time of the node associated to VO *i* as:

(2) $$\tau_{i}^{stor}\left(t\right)=\frac{M_{i}^{res}\left(t\right)}{\sum_{k}D_{k}\cdot f_{ik}}$$

with $M_{i}^{res}\left(t\right)$ residual memory expressed in bits, and $D_{k}$ amount of data to be stored for task *k*. Note that residual memory can change over time, not only because of its usage, but also because its stored data can be moved to another location.

#### 4.1.3. Processor

The time needed to perform a task is in inverse proportion to the processing speed of the node that is performing it, and in direct proportion to the number of instructions required by the task. Calling $t_{ik}^{exec}$ the time needed by the node associated to VO *i* to perform task *k*, it can be stated that

(3) $$t_{ik}^{exec}=\frac{N_{k}^{instr}}{S_{i}^{proc}}$$

where $N_{k}^{instr}$ is the number of instructions that need to be processed to perform task *k*, and $S_{i}^{proc}$ is the processing speed for the node associated to VO *i*. If task *k* is performed at a frequency $f_{ik}\left(t\right)$, this means that the processor of the node associated to VO *i* will be busy for a ratio of time equivalent to

(4) $$\theta_{ik}^{proc}\left(t\right)=t_{ik}^{exec}\cdot f_{ik}\left(t\right)$$

Generalising for all the tasks performed by *i*, we define the total processor occupancy as:

(5) $$\Theta_{i}^{proc}\left(t\right)=\sum_{k}t_{ik}^{exec}\cdot f_{ik}\left(t\right)$$

which is the ratio of time for which the processor is busy, considering all the tasks.

#### 4.1.4. Bandwidth

Analogously to the analysis made for the processor occupancy, and considering that the bandwidth needed by the node associated to VO *i* to transmit the output data for task *k* is proportional to the $D_{k}$ bits of data to transmit and to the frequency $f_{ik}\left(t\right)$ at which they are transmitted, we define the bandwidth occupancy as:

(6) $$\Theta_{i}^{BW}\left(t\right)=\frac{\sum_{k}D_{k}\cdot f_{ik}\left(t\right)}{B_{i}^{tot}}$$

where $B_{i}^{tot}$ is the available bandwidth for *i*.

### 4.2. Consensus-Based Resource Allocation Optimisation

The resource optimisation strategy proposed in this paper relies on a consensus-based algorithm, where VOs decide the amount of resources to allocate to a task, according to the constraints requested by the higher layers.

We generalise the equation that describes the use of a resource by VO *i* as:

(7) $$\Theta_{i}\left(t\right)=\sum_{k}\alpha_{ik}\cdot f_{ik}\left(t\right),\;\mathrm{with}\alpha_{ik}\left(t\right)=\left\{\begin{array}{cc} E_{ik}^{c}/E_{i}^{res}\left(t\right), & \mathrm{if}\;\Theta_{i}\left(t\right)=1/\tau_{i}^{lftm}\left(t\right),\\ D_{k}/M_{i}^{res}\left(t\right), & \mathrm{if}\;\Theta_{i}\left(t\right)=1/\tau_{i}^{stor}\left(t\right),\\ t_{ik}^{exec}, & \mathrm{if}\;\Theta_{i}\left(t\right)=\Theta_{i}^{proc}\left(t\right),\\ D_{k}/B_{i}^{tot}, & \mathrm{if}\;\Theta_{i}\left(t\right)=\Theta_{i}^{BW}\left(t\right). \end{array}\right.$$

From the analysis carried out in the previous section, it is evident that optimising the use of the resources belonging to the nodes involved in the system entails adjusting the use that VOs make of them, so that nodes are not overloaded. In other words, the frequency at which each node performs the tasks assigned to it needs to be adjusted so that the effort put by each node to contribute to the execution of tasks needed by the system is equally shared among all of them. This means that, given two VOs *i* and *j* that received an activation request for task *k*, at time $t_{c}$ when the algorithm converges, $\Theta_{i}\left(t_{c}\right)=\Theta_{j}\left(t_{c}\right)$. Therefore,

(8) $$\sum_{k}\alpha_{ik}\left(t_{c}\right)f_{ik}\left(t_{c}\right)=\sum_{k}\alpha_{jk}\left(t_{c}\right)f_{jk}\left(t_{c}\right)$$

Defining the total amount of resource usage contributions with the exception of task *k* as $\delta_{ik}\left(t\right)=\sum_{l\neq k}\alpha_{il}\left(t\right)\cdot f_{il}\left(t\right)$, it follows that

(9) $$f_{jk}\left(t_{c}\right)=\frac{\alpha_{ik}\left(t_{c}\right)}{\alpha_{jk}\left(t_{c}\right)}\cdot f_{ik}\left(t_{c}\right)+\frac{\delta_{ik}\left(t_{c}\right)-\delta_{jk}\left(t_{c}\right)}{\alpha_{jk}\left(t_{c}\right)}$$

According to accuracy constraints provided by the higher layers, the collaborative completion of a task is required to be performed at a reference frequency $F_{k}^{ref}=\sum_{j}f_{jk}\left(t_{c}\right)$. Using Equation (9) in this identity, after some simple computations and multiplying and dividing by the number $N_{k}$ of VOs involved in task *k*, we obtain

(10) $$\alpha_{ik}\left(t_{c}\right)\cdot f_{ik}\left(t_{c}\right)=\frac{{\bar{\varphi }}_{k}}{{\bar{\beta }}_{k}\left(t_{c}\right)}+\frac{{\bar{\gamma }}_{k}\left(t_{c}\right)}{{\bar{\beta }}_{k}\left(t_{c}\right)}-\delta_{ik}\left(t_{c}\right)$$

with

$$\begin{array}{cc} {\bar{\varphi }}_{k} & =\frac{F_{k}^{ref}}{N_{k}}\\ {\bar{\beta }}_{k}\left(t_{c}\right) & =\frac{1}{N_{k}}\cdot \sum_{j}\frac{1}{\alpha_{jk}\left(t_{c}\right)}\\ {\bar{\gamma }}_{k}\left(t_{c}\right) & =\frac{1}{N_{k}}\cdot \sum_{j}\frac{\delta_{jk}\left(t_{c}\right)}{\alpha_{jk}\left(t_{c}\right)} \end{array}$$

It is easy to notice that they represent mean values evaluated over all the VOs that are able to perform task *k*. This fact, along with the consideration that nodes that are assigned to the same task are usually located close to each other, and thus they can communicate directly without passing through the cloud, leads to the conclusion that their value can be estimated in a distributed way using an average consensus algorithm. We suppose to have a system where nodes may not be connected during the whole convergence process. For this reason, in this paper, the consensus algorithm proposed in [24], which is robust against topology changes, is used. Since variations of *α* and *δ* are negligible over the time needed by the algorithm to converge (as it will be clarified in the experiments), in the following, we consider them constant and omit their dependence from time. Nevertheless, if substantial variations of them are experienced, the algorithm needs to start again.

### 4.3. Resource Allocation Optimisation Algorithm

As soon as VO *i* receives an activation request for task *k*, it initialises its local values $\varphi_{ik}=\varphi_{ik}^{0}$, $\beta_{ik}=\beta_{ik}^{0}$ and $\gamma_{ik}=\gamma_{ik}^{0}$. As far as $\varphi_{ik}$ is concerned, only one VO receives the reference frequency $F_{k}^{ref}$ from the VO level and sets $\varphi_{ik}^{0}$ to it. The other VOs set it to 0. The initial local values are set as follows:

(11) $$\begin{array}{cc} \varphi_{ik}^{0} & =\left\{\begin{array}{cc} F_{k}^{ref}, & \mathrm{if}\;F_{k}^{ref}\;\mathrm{is}\;\mathrm{given}\\ 0, & \mathrm{otherwise} \end{array}\right.\\ \beta_{ik}^{0} & =\frac{1}{\alpha_{ik}},\;\;\gamma_{ik}^{0}=\frac{\delta_{ik}}{\alpha_{ik}} \end{array}$$

and starts the consensus with its neighbours. VO *j* is a neighbour for VO *i* if and only if they are directly connected, i.e., they are one-hop far from each other (note that, since VOs act at a logical level, in order for them to be one-hop far it is not necessary that their related RWOs are directly connected, but it is sufficient that their VOs are logically directly connected). At each step of the consensus algorithm, i.e., whenever VO *i* receives an update from one of its neighbours $j\in N_{i}$, where $N_{i}$ is the set of neighbours of node *i*, it computes the following updates:

(12a) $$\begin{array}{cc} \varphi_{ik}^{+} & =\varphi_{ik}-\lambda_{1}^{\varphi }\sum_{j\in N_{i}}\left(\varphi_{ik}-\varphi_{jk}\right)-\lambda_{2}^{\varphi }\sum_{j\in N_{i}}\mathrm{sgn}\left(\varphi_{ik}-\varphi_{jk}\right), \end{array}$$

(12b) $$\begin{array}{cc} \beta_{ik}^{+} & =\beta_{ik}-\lambda_{1}^{\beta }\sum_{j\in N_{i}}\left(\beta_{ik}-\beta_{jk}\right)-\lambda_{2}^{\beta }\sum_{j\in N_{i}}\mathrm{sgn}\left(\beta_{ik}-\beta_{jk}\right), \end{array}$$

(12c) $$\begin{array}{cc} \gamma_{ik}^{+} & =\gamma_{ik}-\lambda_{1}^{\gamma }\sum_{j\in N_{i}}\left(\gamma_{ik}-\gamma_{jk}\right)-\lambda_{2}^{\gamma }\sum_{j\in N_{i}}\mathrm{sgn}\left(\gamma_{ik}-\gamma_{jk}\right), \end{array}$$

(12d) $$\begin{array}{cc} \Theta_{i}^{+} & =\frac{\varphi_{ik}^{+}+\gamma_{ik}^{+}}{\beta_{ik}^{+}}\;\;f_{ik}^{+}=\frac{1}{\alpha_{ik}}\cdot \left(\Theta_{i}^{+}-\delta_{ik}\right), \end{array}$$

where $\lambda_{1}^{\varphi }$, $\lambda_{1}^{\beta }$, $\lambda_{1}^{\gamma }$, $\lambda_{2}^{\varphi }$, $\lambda_{2}^{\beta }$, and $\lambda_{2}^{\gamma }$ are tuning parameters that affect the convergence time and steady-state accuracy [24], and that will be better explained in the following subsection. If $f_{ik}^{+}>0$ and if its value has changed after the update, the VO sends the updated value of $\varphi_{ik}^{+}$, $\beta_{ik}^{+}$ and $\gamma_{ik}^{+}$ to its neighbours. It may happen that $f_{ik}^{+}\leq 0$. In this case, the VO cannot participate into executing task *k*. Therefore, it sets $f_{ik}$ to 0 and informs its neighbours, which restart the consensus process. The algorithm can be considered converged when $f_{ik}$ does not change after the updates.

### 4.4. Convergence Time and Steady-State Accuracy

The proposed consensus protocol represents a discrete-time application of the finite-time discontinuous average-based consensus algorithm discussed, respectively, in [25] for a network of connected continuous time integrators and in [26] for networks of perturbed, and possibly switching, spanned-tree topologies. It follows that, as long as the stability of the linear part of the problem in Equation (12) is preserved, the convergent properties discussed in [25,26] are in force. Thus, from [26,27], it is straightforward to derive that

(13) $$\begin{array}{c} 0\leq \lambda_{1}^{\varphi },\lambda_{1}^{\beta },\lambda_{1}^{\gamma }\leq \frac{1}{\max_{i}\left|N_{i}\right|} \end{array}$$

where $|N_{i}|$ denotes the number of neighbours of node *i*. Note that Equation (13) derives straightforward from considerations on discrete-time consensus and Perron matrices, which, however, go beyond the scope of this research. Further details can be found in [27,28]. If condition (13) holds, then the results of [26] are directly applicable for the characterization of the convergence properties of the discrete-time collective multi-agent dynamic in Equation (12).

**Figure 5:**
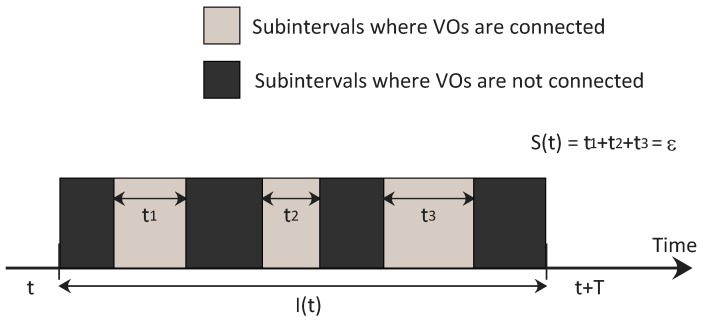
Communication constraints for the consensus algorithm to converge [25].

Thus, following Assumption 1 of [25], let *ε* and *T*, with ε≤T being two positive constants, where *T* defines the length of a receding horizon time interval I(t)=(t,t+T), and *ε* is the total length of the subinterval S(t)≤I(t) given by the union of the subintervals during which the network is connected (see Figure 5 for a graphical explanation of the interval I(t) and S(t)), it results that, for the problem in Equation (12), consensus will be reached in finite time if

(14) $$\lambda_{2}^{\varphi },\lambda_{2}^{\beta },\lambda_{2}^{\gamma }\geq 2\cdot \frac{T}{\varepsilon }+\mu^{2}$$

with μ≠0. If condition (14) holds, the convergence is reached with accuracy Γ after, at most, a transient time $t_{r}$ that is proportional to the maximum deviation of the agents’ states at the start-up (i.e., when *t* = 0) of the algorithms

(15) $$\begin{array}{cc} & t_{r}\leq \left(\frac{T}{\varepsilon \mu^{2}}\right)\cdot \max_{i,j}\left|x_{i}^{0}-x_{j}^{0}\right|\\ & \Gamma =2\cdot (T-\varepsilon )+\xi \end{array}$$

where ξ>0 is an arbitrary infinitesimally small parameter, and $x_{i}^{0},x_{j}^{0}$ are the initial values for VOs *i* and *j* of the generic consensus variables, which, in our case, are those specified by Equation (11).

As it is specified in [24], the tuning parameters of the update functions need to be set to:

(16) $$\begin{array}{cc} & \lambda_{1}^{\varphi },\lambda_{1}^{\beta },\lambda_{1}^{\gamma }\geq 0\\ & \lambda_{2}^{\varphi },\lambda_{2}^{\beta },\lambda_{2}^{\gamma }\geq \frac{2T\cdot \Pi }{\varepsilon }+\mu^{2},\;\mu \neq 0 \end{array}$$

where: *ε* and *T* are positive constants, and *T* is a horizon time interval such that the involved VOs are connected at least for an *ε* amount of time (ε≤T); and Π and *μ* are weight parameters. Appropriately choosing the tuning parameters affects the accuracy of the solution of the algorithm, as well as the convergence time, as follows:

(17) $$\begin{array}{c} \mathrm{Accuracy}=[2\cdot (T-\varepsilon )+\xi ]\cdot \Pi \\ \mathrm{Convergence}\;\mathrm{time}\leq \left(\frac{T}{\varepsilon \mu^{2}}\right)\cdot \max_{i,j}\left|x_{i}^{0}-x_{j}^{0}\right| \end{array}$$

where ξ>0 is an arbitrary infinitesimally small parameter, and $x_{i}^{0},x_{j}^{0}$ are the initial values for VOs *i* and *j* of the generic consensus variables, which, in our case, are those specified by Equation (11). These conditions ensure that the algorithm converges to a solution in a finite time with an accuracy that depends on the tuning parameters.

Supposing that T=ε, i.e., the VOs are always connected during the consensus process:

(18) $$\begin{array}{cc} & t_{r}\leq \left(\frac{1}{\mu^{2}}\right)\cdot \max_{i,j}\left|x_{i}^{0}-x_{j}^{0}\right|\\ & \Gamma =\xi . \end{array}$$

## 5. The Proposed IoT System

In this section, the whole IoT resource allocation system proposed in this paper is described. Algorithm 1 provides the pseudo-code for the whole process. As soon as the service level receives a request for a task, it translates it into computer language, generating the query $Q_{k}$, which is sent to the VO level. Based on $Q_{k}$, the VO level finds the VO Information Model that best fits the characteristics required by $Q_{k}$. The VO level than starts a search for the set $S_{k}$ of VO instances that correspond to the required VO Information Model, i.e., the set of VOs that can respond to the query. Then, the VO level selects one of the VOs to whom forwarding the request, i.e., the candidate VO $VO_{0}$. Since the candidate VO has to perform some additional operations with respect to the other VOs, the VO level tries to choose the one that is likely to have more resources. For this reason, if in $S_{k}$ there is at least one VO that is located in the cloud, the candidate VO is chosen randomly among them; otherwise, if there is at least one VO that is located in an intermediate gateway, the candidate VO is chosen to be the one located in the closest gateway; if all the VOs are located remotely, the candidate VO is chosen to be the closest one. The VO level sends to the candidate VO a message $M_{k}$, including the reference frequency, the set of VOs, the resource to be optimally allocated and the time interval $T_{k}$ during which the task has to be continuously performed: $M_{k}=\left\{F_{k}^{ref},S_{k},resource,T_{k}\right\}$.
**Algorithm 1** The Proposed IoT System1:The service level receives a request for task *k*2:The service level translates the task request into query $Q_{k}$3:The service level sends $Q_{k}$ to the VO level4:The VO level finds the appropriate VO Information Model to respond to $Q_{k}$5:The VO level finds the set $S_{k}$ of VOs corresponding to the required VO Information Model6:**if** at least one VO $\in S_{k}$ is in the cloud **then**7:  Set it as $VO_{0}$8:**else if** at least one VO $\in S_{k}$ is in an intermediate gateway **then**9:  Set the VO in the closest intermediate gateway as $VO_{0}$10:**else**11:  Set the closest VO as $VO_{0}$12:**end if**13:The VO level sends message $M_{k}$ to $VO_{0}$14:$VO_{0}$ evaluates Equation (20)15:**if** Equation (20) is **false then**16:  $VO_{0}$ assigns $f_{ik}=F_{k}^{ref}/\left|S_{k}\right|$, $\forall i\in S_{k}$17:**else**18:  $VO_{0}$ sends the activation request and initialization message for task *k* to the nodes in $S_{k}$19:  **for each** $i\in S_{k}$ **do**20:    **if** An initialization message is received **then**21:      Initialize $\varphi_{ik}$, $\beta_{ik}$ and $\gamma_{ik}$ values according to Equation (11)22:    **end if**23:    **if** An update message is received **then**24:      Compute $\varphi_{ik}^{+}$, $\beta_{ik}^{+}$, $\gamma_{ik}^{+}$ and $f_{ik}^{+}$ values according to Equation (12)25:      **if** $f_{ik}^{+}$>0 **then**26:        **if** $f_{ik}^{+}\neq f_{ik}$ **then**27:          *i* sends $\varphi_{ik}^{+}$, $\beta_{ik}^{+}$ and $\gamma_{ik}^{+}$ values to all $j\in N_{i}$28:        **end if**29:      **else**30:        *i* sets $f_{ik}=0$ and sends an initialization message to all $j\in N_{i}$31:      **end if**32:    **end** **if**33:  **end for**34:**end if**35:**if** Any substantial change is experiences by VOs **then**36:  Go to step 1437:**end if**

After receiving the request from the VO level, the candidate VO has to choose whether or not the consensus algorithm is convenient to be started. Indeed, since the consensus process requires a certain amount of resources, before proceeding with it, it is important to evaluate if it is convenient to the system, i.e., if the amount of resources saved thanks to consensus is higher than the amount of resources needed to reach a consensus. It is trivial to demonstrate that, if $T_{k}=\infty$, i.e., task *k*’s duration is not specified by the request, the consensus execution is always convenient. If $T_{k}$ is limited, the candidate VO has to evaluate how much the consensus algorithm costs in terms of resources, with respect to the requested task. We call $\alpha_{i}^{cons}$ the amount of resource consumed to perform a single step of the consensus algorithm, i.e., the value of $\alpha_{ik}$ computed according to Equation (7) considering not a single execution of task *k*, but a single execution of a step of the consensus algorithm. Let ${\bar{N}}^{step}$ be the average number of steps required by consensus to converge. Performing consensus is convenient if the following condition is satisfied:

(19) $$\alpha_{i}^{cons}\cdot {\bar{N}}^{step}\ll \alpha_{ik}\cdot f_{ik}\cdot T_{k}.$$

Approximating $f_{ik}$ with $F_{k}^{ref}/\left|S_{k}\right|$, where $|S_{k}|$ is the number of VOs in $S_{k}$, it is possible to approximate the condition above as follows:

(20) $$\alpha_{i}^{cons}<\alpha_{ik}\cdot \frac{F_{k}^{ref}}{|S_{k}|}\cdot \frac{T_{k}}{\Lambda \cdot {\bar{N}}^{step}},$$

where Λ is an arbitrarily high design parameter. Considering, for example, 10 VOs, $\alpha_{i}^{cons}=\alpha_{ik}$, $F_{k}^{ref}=0.1$ Hz, Λ=20 and ${\bar{N}}^{step}=7$ (which, as shown in Section 6, is a reasonable value), the condition in Equation (20) is met for $T_{k}>3.8$ h. If the amount of saved resources is not expected to be sufficient, the process is not started at all, and frequencies are assigned to the nodes in $S_{k}$ according to a static assignment, e.g., they are set to $F_{k}^{ref}/\left|S_{k}\right|$ for each node. Otherwise, the candidate VO sends the activation request and initialization message for task *k* to the nodes in $S_{k}$ and the algorithm described in Section 4.3 is started. Note that it is not necessary that nodes in $S_{k}$ are directly connected: it is sufficient that their VOs are connected (either physically or logically) by a limited number of hops).

After the frequency values have been assigned to the appropriate VOs, the task is performed by them for the amount of time that was required in the original request. If any substantial changes were experienced by VOs (e.g., depletion of the allocated resource, change in position of one of the VOs, a new VO with the appropriate characteristics is detected), the candidate VO is informed of them, and the algorithm starts again from the step where the candidate VO evaluates Equation (20).

## 6. Experiments

The proposed algorithm has been implemented to run in the Arduino Mega 2560 (Arduino SRL, Torino, Italy) [29] device, whose microcontroller is an ATmega 2560. The local network was created through XBee S1 802.15.4 modules, by Digi International (Digi International Inc., Minnetonka, MN, USA) [30]. These modules use the IEEE 802.15.4 networking protocol for fast point-to-multipoint or peer-to-peer networking. The XBee modules are ideal for low-power and low-cost applications. The XBee modules have been connected to Arduino via serial port, using Xbee USB serial adapters by DF Robot (DFRobot, Shanghai, China) [31]. Tests were performed considering up to 10 real devices participating in the optimisation process for the allocation of up to 10 tasks. Devices are connected in a mesh fashion, i.e., they are all connected, and neither disconnections nor noise is experienced. Therefore, tuning parameter are set to $\lambda_{1}^{\varphi },\lambda_{1}^{\beta },\lambda_{1}^{\gamma }=1/\left|S_{k}\right|$ (remind that |$S_{k}$| is the number of VOs involved in task *k*) and $\lambda_{2}^{\varphi },\lambda_{2}^{\beta },\lambda_{2}^{\gamma }=0$. We supposed that tasks with different complexities are assigned to nodes one at a time. Nodes have a residual energy ranging from 2 to 3 kJ. We also supposed to know the energy consumption value associated to each task at each node. According to it, energy consumption values for a single execution of each task are assigned randomly to the nodes in the ranges defined in Table 1. As a term for comparison, typical energy consumption values to transmit data using XBee modules are ∼0.3 mJ/byte [30,32], while approximately 7 μJ are needed, on a typical board, to execute a simple application such as the average of five numbers [33].

**Figure 6:**
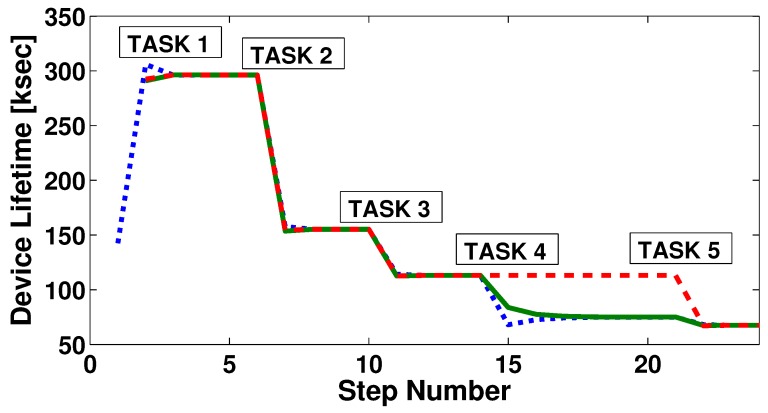
Example plot for algorithm convergence.

Figure 6 shows in an explanatory example how three devices reach consensus for five different tasks. Each line style is associated with a different device. Whenever a new task is activated, the devices that can perform that task initiate the consensus process. The initialisation instants correspond to the peaks in the figures and are marked by the respective label. It is possible to see how, for each task, the convergence is reached in just a few steps. On average, the algorithm takes only less than seven steps per task to converge. For each task activation, the lifetime values of the three devices converge, as the frequency of execution is distributed in an optimised manner to reach the reference frequency. In the example, task 4 can be performed by only two devices out of three. Thus, only two devices take charge of the workload related to task 4, and their lifetime value converges toward a lower value than that of the other device. After the algorithm has run for task 4, it could be run again for the tasks whose frequency has already been assigned, in order for the devices to equally redistribute the workload and reach the same lifetime value again. Nevertheless, we believe that the benefit introduced by this process would not be enough, especially considering that the following tasks will have the same result of making the devices converge to the same lifetime. Indeed, in the example, once the fifth task is activated, frequencies are divided one more time and devices reach the same lifetime value again.

To evaluate the performance of the algorithm, we compared three different approaches:

- Network lifetime achieved using the proposed algorithm (indicated with label *optτ*);
- Network lifetime when each task is entirely assigned to the node with the lowest energy consumption value related to that task (label *minE*);
- Network lifetime when the task’s reference frequency $F_{k}^{ref}$ equally divided by the number of devices available to run it (label *eqF*).

**Figure 7:**
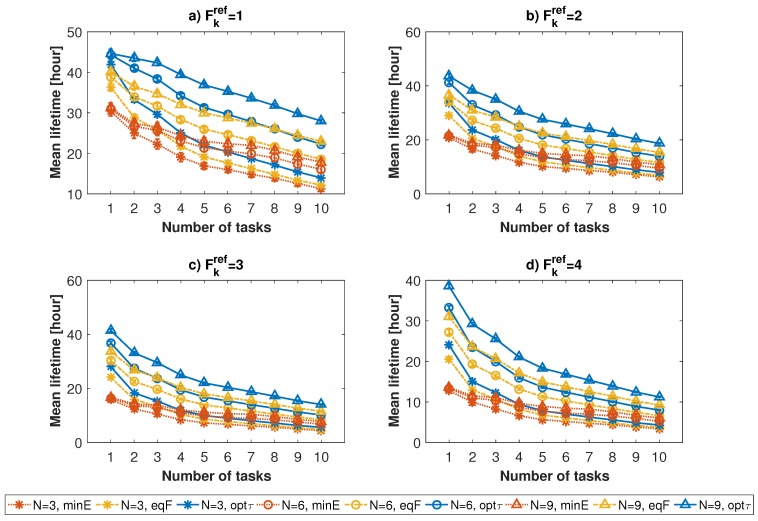
Average values of network lifetimes when the number of tasks increases, for a number of available nodes equal to 3 (star marker), 6 (circle marker) and 9 (triangle marker). Results are shown for reference frequency values equal to 1 (**a**); 2 (**b**); 3 (**c**) and 4 (**d**).

**Figure 8:**
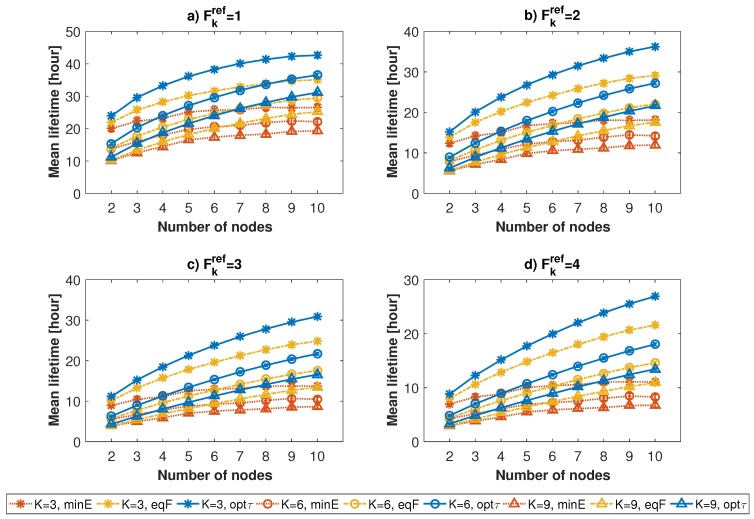
Average values of network lifetimes when the number of nodes increases, for a number of assigned tasks equal to 3 (star marker), 6 (circle marker) and 9 (triangle marker). Results are shown for reference frequency values equal to 1 (**a**); 2 (**b**); 3 (**c**) and 4 (**d**).

Figure 7 and Figure 8 show the average network lifetime and related confidence interval, using the three different approaches, for different numbers of assigned tasks and nodes (indicated, respectively, with labels *K* and *N*). The graphs show that the optimal resource allocation algorithm always outperforms the other approaches, especially with respect to *minE*. The gap is particularly evident when the amount of available resources is higher than that of required resources, i.e., when the number of nodes is high, or when the number of assigned tasks and reference frequency are low. This is motivated by the fact that, with the non-optimized solutions, if the number of tasks is lower than the number of involved nodes, the probability to have an unfair distribution of energy among nodes is higher with respect to that of a high number of tasks. Therefore, the higher the amount of available resources, the better the behaviour of the resource allocation algorithm. The lifetime improvement of the optimal resource allocation algorithm goes from 12% to 60.3% for the *minE* approach and from 6.5% to 20.8% for the *eqF* approach.

**Figure 9:**
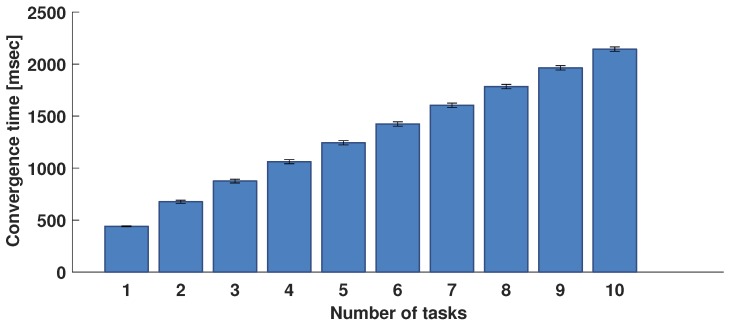
Average values of convergence time.

The behaviour of the algorithm was also evaluated from the time performance point of view. The convergence times measured during the testing phase and related confidence interval are shown in Figure 9 as a function of the number of tasks to be assigned. It goes from 440 ms when only two tasks are assigned to 2.14 s when 10 tasks are assigned, with an average convergence time of 214 ms per task.

## 7. Conclusions

We proposed the use of VOs to control and manage the heterogeneous resource-constrained objects that characterise the IoT, so as to find those that are able to perform some given tasks ensuring the required QoI. We then introduced a consensus-based algorithm where the resources of these objects, specifically their residual battery charge, are assigned to the execution of tasks so that the workload is distributed in a fair way. Tests on real devices showed that we can reach an average lifetime improvement of more than 20% using up to 10 devices, which decreases when the amount of available resources decreases.

## Figures and Tables

**Table 1:** Energy consumption values per task’s single execution.

**Task ID**	**Task 1**	**Task 2**	**Task 3**	**Task 4**	**Task 5**
$E_{\mathrm{ik}}^{c}$ **value [mJ]**	6.82÷12.27	7.50÷13.49	9.70÷17.46	6.17÷11.11	5.11÷9.20
**Task ID**	**Task 6**	**Task 7**	**Task 8**	**Task 9**	**Task 10**
$E_{\mathrm{ik}}^{c}$ **value [mJ]**	6.51÷11.71	8.49÷15.28	9.13÷16.43	5.68÷10.23	9.07÷16.33

## References

[1] Bisdikian C., Kaplan L.M., Srivastava M.B. (2013). On the quality and value of information in sensor networks. ACM Trans. Sens. Netw..

[2] Wang W., De S., Toenjes R., Reetz E., Moessner K. A comprehensive ontology for knowledge representation in the internet of things. Proceedings of the 2012 IEEE 11th International Conference on Trust, Security and Privacy in Computing and Communications (TrustCom).

[3] Gubbi J., Buyya R., Marusic S., Palaniswami M. (2013). Internet of Things (IoT): A vision, architectural elements, and future directions. Future Gener. Comput. Syst..

[4] Nitti M., Pilloni V., Colistra G., Atzori L. (2015). The Virtual Object as a Major Element of the Internet of Things: A Survey. IEEE Commun. Surv. Tutor..

[5] Atzori L., Iera A., Morabito G. (2010). The internet of things: A survey. Comput. Netw..

[6] Barnaghi P., Wang W., Henson C., Taylor K. (2012). Semantics for the Internet of Things: Early progress and back to the future. IGI Glob..

[7] Gluhak A., Krco S., Nati M., Pfisterer D., Mitton N., Razafindralambo T. (2011). A survey on facilities for experimental internet of things research. IEEE Commun. Mag..

[8] Sehgal V.K., Patrick A., Rajpoot L. A comparative study of cyber physical cloud, cloud of sensors and internet of things: Their ideology, similarities and differences. Proceedings of the 2014 IEEE International Advance Computing Conference (IACC).

[9] Khan I., Belqasmi F., Glitho R., Crespi N., Morrow M., Polakos P. (2015). Wireless sensor network virtualization: A survey. IEEE Commun. Surv. Tutor..

[10] Khan I., Errounda F.Z., Yangui S., Glitho R., Crespi N. Getting Virtualized Wireless Sensor Networks’ IaaS Ready for PaaS. Proceedings of the 2015 IEEE International Conference on Distributed Computing in Sensor Systems.

[11] Khan I., Jafrin R., Errounda F.Z., Glitho R., Crespi N., Morrow M., Polakos P. A data annotation architecture for semantic applications in virtualized wireless sensor networks. Proceedings of the 2015 IFIP/IEEE International Symposium on Integrated Network Management (IM).

[12] Pacual-Espada J., Sanjuan-Martinez O., G-Bustelo B.C.P., Lovelle J.M.C. (2011). Virtual objects on the Internet of things. Int. J. Interact. Multimed. Artif. Intell..

[13] Suciu G., Vulpe A., Halunga S., Fratu O., Todoran G., Suciu V. Smart cities built on resilient cloud computing and secure internet of things. Proceedings of the 2013 19th IEEE International Conference on Control Systems and Computer Science (CSCS).

[14] Farris I., Militano L., Nitti M., Atzori L., Iera A. Federated edge-assisted mobile clouds for service provisioning in heterogeneous IoT environments. Proceedings of the 2015 IEEE 2nd World Forum on Internet of Things (WF-IoT).

[15] Pilloni V., Navaratnam P., Vural S., Atzori L., Tafazolli R. Cooperative task assignment for distributed deployment of applications in WSNs. Proceedings of the 2013 IEEE International Conference on Communications (ICC).

[16] Shen Y., Ju H. Energy-Efficient Task Assignment Based on Entropy Theory and Particle Swarm Optimization Algorithm for Wireless Sensor Networks. Proceedings of the 2011 IEEE/ACM International Conference on Green Computing and Communications (GreenCom).

[17] Guinard D., Trifa V., Mattern F., Wilde E. (2011). From the internet of things to the web of things: Resource-oriented architecture and best practices. Architecting the Internet of Things.

[18] Silverajan B., Harju J. Developing network software and communications protocols towards the internet of things. Proceedings of the ACM Fourth International ICST Conference on Communication System Software and Middleware.

[19] Colistra G., Pilloni V., Atzori L. (2014). The problem of task allocation in the Internet of Things and the consensus-based approach. Comput. Netw..

[20] Vlacheas P., Giaffreda R., Stavroulaki V., Kelaidonis D., Foteinos V., Poulios G., Demestichas P., Somov A., Biswas A.R., Moessner K. (2013). Enabling smart cities through a cognitive management framework for the internet of things. IEEE Commun. Mag..

[21] iCore Project (2015). iCore: Empowering IoT through Cognitive Tecnologies. http://www.iot-icore.eu/about-icore.

[22] Bonomi F., Milito R., Zhu J., Addepalli S. Fog computing and its role in the internet of things. Proceedings of the ACM First Edition of the MCC Workshop on Mobile Cloud Computing.

[23] Yun Y., Xia Y., Behdani B., Smith J.C. (2013). Distributed algorithm for lifetime maximization in a delay-tolerant wireless sensor network with a mobile sink. IEEE Trans. Mob. Comput..

[24] Pilloni A., Franceschelli M., Pisano A., Usai E. Recent advances in sliding-mode based consensus strategies. Proceedings of the 2014 13th IEEE International Workshop on Variable Structure Systems (VSS).

[25] Franceschelli M., Pilloni A., Pisano A., Giua A., Usai E. Finite-time consensus with disturbance attenuation for directed switching network topologies by discontinuous local interactions. Proceedings of the 52nd IEEE Conference on Decision and Control.

[26] Jiang F., Wang L. (2011). Finite-time weighted average consensus with respect to a monotonic function and its application. Syst. Control Lett..

[27] Olfati-Saber R., Fax J.A., Murray R.M. (2007). Consensus and cooperation in networked multi-agent systems. Proc. IEEE.

[28] Godsil C., Royle G.F. (2013). Algebraic Graph Theory.

[29] Arduino (2015). Arduino Mega 2560. https://www.arduino.cc/en/Main/ArduinoBoardMega2560.

[30] D.I., Inc. (2015). Xbee S1. http://www.digi.com/products/xbee-rf-solutions/modules/xbee-series1-module.

[31] Robot D. (2015). Xbee S1. http://www.dfrobot.com/index.php?route=product/product&product_id=72#.Vd7g1fntlHx.

[32] Liu M., Cao J., Chen G., Wang X. (2009). An energy-aware routing protocol in wireless sensor networks. Sensors.

[33] Dâmaso A., Freitas D., Rosa N., Silva B., Maciel P. (2013). Evaluating the power consumption of wireless sensor network applications using models. Sensors.

